# Leuprorelin Acetate Long-Lasting Effects on GnRH Receptors of Prostate Cancer Cells: An Atomic Force Microscopy Study of Agonist/Receptor Interaction

**DOI:** 10.1371/journal.pone.0052530

**Published:** 2013-01-09

**Authors:** Gina Lama, Massimiliano Papi, Cristiana Angelucci, Giuseppe Maulucci, Gigliola Sica, Marco De Spirito

**Affiliations:** 1 Istituto di Istologia ed Embriologia, Facoltà di Medicina e Chirurgia “A. Gemelli”, Università Cattolica del Sacro Cuore, Roma, Italy; 2 Istituto di Fisica, Facoltà di Medicina e Chirurgia “A. Gemelli”, Università Cattolica del Sacro Cuore, Roma, Italy; II Università di Napoli, Italy

## Abstract

High cell-surface GnRH receptor (GnRH-R) levels have been shown to have a major influence on the extent of GnRH agonist-mediated tumor growth inhibition. The ability of the GnRH agonist leuprorelin acetate (LA) to induce a post-transcriptional upregulation of GnRH-R at the plasma membrane of androgen-sensitive (LNCaP) and -insensitive (PC-3) prostate cancer (PCa) cells has been previously demonstrated by Western blotting. Here we performed single molecule force spectroscopy by using Atomic Force Microscopy (AFM), which has proven to be a powerful tool allowing for investigation of living cell surface biological features, such as the so far unclear GnRH agonist/receptor interaction. Thus, in the hormone-insensitive PC-3 cells, we characterized the strength of the LA-receptor binding, and the amount and distribution of the functional receptor molecules on the cell surface. The effect of a long and continuous treatment (up to 30 days) with the agonist (10^−11^ and 10^−6^ M) on the same parameters was also investigated. A GnRH-R increase was observed, reaching the maximum (∼80%) after 30 days of treatment with the highest dose of LA (10^−6^ M). The analogue-induced increase in GnRH-R was also demonstrated by Western blotting. In addition, two different receptor bound strengths were detected by AFM, which suggests the existence of two GnRH-R classes. A homogeneous distribution of the unbinding events has been found on untreated and treated PC-3 cell surfaces. The persistence of high receptor levels at the membrane of these living cells may warrant the maintenance of the response to LA also in androgen-unresponsive PCa. Moreover, the determination of ligand/receptor bond strength could shed light on the poorly understood event of LA/GnRH-R interaction and/or address structural/chemical agonist optimizations.

## Introduction

The gonadotropin-releasing hormone (GnRH), a decapeptide secreted in a pulsatile fashion by hypothalamic neurons, controls gonadotropin synthesis and release by activating receptors (type I GnRH-R) expressed on anterior pituitary cells. The down-regulation and desensitization of these hypophyseal receptors by continuous administration of GnRH agonistic analogues represent the rationale for the clinical use of these hormones in the therapy of endocrine-related cancers since it leads to gonadal steroid suppression [1]–[3]. The finding of GnRH/GnRH-R expression in these tumors, as well as in nonmalignant tissues [4]–[7], disclosed the possibility for cells of extrapituitary tissues to be directly affected by GnRH analogues. Different studies demonstrated the inhibitory effect of GnRH agonists on the growth of various neoplasms including prostate cancer (PCa) cells [6], [8]–[14]. Nevertheless, some authors reported that GnRH agonists are ineffective when used alone while counteract or even suppress hormone- or growth factor-stimulated cell proliferation [15]–[19]. In addition, they demonstrated that these compounds are able to modulate PSA expression as well as the expression of several genes/proteins regulating growth and differentiation, apoptosis or cell/cell adhesion [20]–[23]. More recently, the effects of the GnRH agonistic analogue leuprorelin acetate (LA) on the expression of GnRH-R were investigated by Western blotting in two human PCa cell lines: the androgen-sensitive, well-differentiated and low invasive LNCaP cells and the androgen-insensitive, poorly differentiated and highly invasive PC-3 cells [24]. In these two models, the analogue at both low and high concentrations is effective in inducing a post-transcriptional enhancement of the receptor expression at the plasma membrane level, after 4, 6 and 12 days of a continuous treatment.

The increase in receptor availability at the cell surface could be a relevant therapeutic issue since it may warrant the maintenance of the response to the agonist therapy [25]. Moreover, it may allow for the development of new therapeutic strategies, which is particularly important for those tumors that either fail to respond or develop resistance to endocrine therapy. In fact, even if it is sorely difficult to predict the PCa cell behaviour *in vivo*, it is presumable that, even in hormone-unresponsive PCa, the agonist administration may provide a beneficial outcome also due to the direct effect.

In the present paper, we performed single molecule force spectroscopy by means of Atomic Force Microscopy (AFM) with the aim of characterizing the strength of the LA-receptor binding, and the amount and distribution of the functional receptor molecules at the androgen-unresponsive PC-3 cell surface. We also demonstrated the effect of a long and continuous treatment with the agonist on the same parameters. AFM indeed allows for the detection of dynamic changes in the mechanical properties of living cell surface [26], [27], as well as measurements in air or fluid of unstained and uncoated biological samples in controlled environments and in a nanoscale resolution [28]–[31], without the requirement of any special treatment that can result in cell destruction or alteration. Moreover, single molecule force spectroscopy offers a unique opportunity to detect molecular recognition between individual ligands and receptors. Since the persistence of high receptor levels at the plasma membrane after a relatively long and continuous treatment with GnRH agonist may improve the efficacy of LA in androgen-unresponsive PCa, we investigated the effect of the agonist at low and high concentrations in PC-3 cells for up to 30 days. Intriguing information on so far unclear GnRH agonist-receptor interaction may derive from the study of the strength to which such binding occurs and address structural/chemical optimizations of the analogue.

## Materials and Methods

### Cell lines and culture conditions

The human hormone-insensitive PC-3 cells [32] were kindly donated by Prof F. Labrie (Laval University, Quebec, Canada). Cells were routinely cultured on 25-cm^2^ cell culture flasks in Dulbecco's modified Eagle's medium (DMEM, Euroclone S.p.A., Milan, Italy), supplemented with 5% foetal bovine serum (FBS, Euroclone), antibiotics (100 IU/ml penicillin, 100 µg/ml streptomycin, Euroclone), 10 mM Hepes buffer solution (Euroclone), 1 mM sodium piruvate (Euroclone) and 2 mM L-glutamine (Euroclone).

The human embryonic kidney cells (HEK293) not expressing any endogenous GnRH-R were kindly provided by Prof R. P. Millar (Medical Research Council Human Reproductive Sciences Unit, The Queen's Medical Research Institute, Edinburgh, UK), who obtained them from American Type Tissue Culture Collection. HEK293 cells stably transfected with GnRH-R (HEK293_[SCL60]_) were a gift from Prof. R. P. Millar and were generated in his laboratory [33]. HEK293 and HEK293_[SCL60]_ cells were used as negative and positive control for GnRH-R expression, respectively. Cells were cultured in DMEM, supplemented with 10% FBS, antibiotics (100 IU/ml penicillin, 100 µg/ml streptomycin), 10 mM Hepes buffer solution, 1 mM sodium piruvate and 4 mM L-glutamine.

All the cell lines were incubated in a humidified atmosphere of 5% CO_2_-95% air at 37°C.

### Cell treatments

The cells were seeded at a density of 25,000 cells/ml of standard culture medium in 40-mm TPP dishes (Techno Plastic Products AG, Trasadingen, Switzerland). Once they adhered to the culture plates (after 24 h), the medium was renewed with DMEM supplemented with 5% charcoal-treated FBS (CH-FBS) and 10^−6^ M or 10^−11^ M LA (kindly donated by Takeda Pharmaceutical Company Limited, Osaka, Japan).

The medium was changed every 48 h, while LA was added daily to the cultures. PC-3 cells were exposed to the analogue for 6, 12, 18, 24 and 30 days.

Every six days, cells were trypsinized, seeded (25,000 cells/ml) into the 40-mm TPP dishes and, once they adhered to the culture plates, treated as described above for a further 6-day period. At the end of each treatment period, the cells were analyzed by AFM. In all the experiments, control cultures were run in parallel.

### Atomic Force Microscopy (AFM)

All the measurements were performed by using an atomic force microscope *Nanowizard II* (JPK Instruments, Berlin, Germany) combined with an optical microscope *Axio Observe* (Zeiss, Oberkochen, Germany). All the acquisitions were fulfilled in a PBS solution (pH = 7.4), under a controlled temperature of 37°C.

### AFM Probe Preparation

In order to investigate specific interactions between LA and GnRH-R on the surface of PC-3 cells, the analogue molecules were immobilized onto AFM tips. Rectangular soft silicon nitride microcantilevers with ultrasharp conical tips with a radius of ∼10 nm coated on both sides with gold (CSC16, MicroMash, San Jose, CA) and calibrated as reported by Papi et al. [34] were used.

Prior to immobilization, microcantilevers were washed in chloroform to remove oils and gross contaminants and then exposed for 20 min to a UV-ozone cleaner in order to remove organic and other oxidizable surface contaminants.

The analogue molecule immobilization procedure, a frequently used method for tip functionalization with a ligand molecule [35], is based on the use of an 8 nm long flexible cross-linker (pyridyl dithio-Polyethyleneglycol-succinimidylpropionate, PDP-PEG-NHS, Polypure). The clean cantilever was immediately immersed in a PDP-PEG-NHS/chloroform solution for 3 h (linker concentration: 2 mg/ml) and then, after two rinsing steps in chloroform and one in PBS, treated with a drop of LA solution (2 mg/ml) overnight.

After functionalization, cantilevers were thoroughly washed three times in buffer solution and then stored at 4°C under sterile conditions for further use in the experiments. Storage time was always <1 week. Any significant change in the LA/GnRH-R interaction was observed during this period. Prior to being used, the spring constant of each cantilever was calibrated by using the thermal method [36].

### Atomic Force Spectroscopy

**Figure 1:**
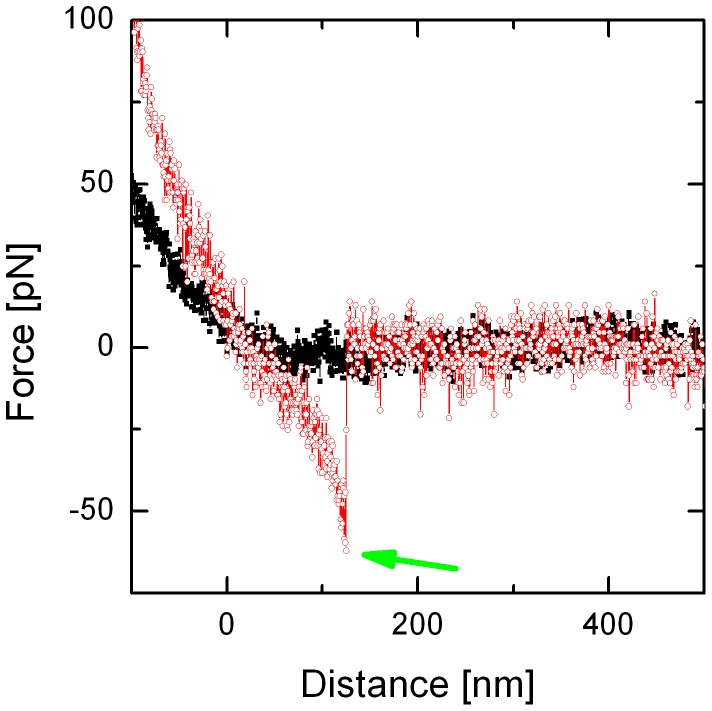
Force-distance curves. Two typical force-distance curves when interactions are not detected (black square) and when interactions are detected (red circle). The unbinding event (or rupture point) is highlighted by means of the green arrow.

To explore single molecular interactions, the dissociation process of a tip-bound ligand from a cell surface-bound receptor was studied by applying a pulling force to the ligand-receptor complex until the binding between ligand and receptor broke. Interaction forces of tip-bound ligands and surface immobilized receptors were measured in force-distance cycles. At a fixed position, the tip was approached to the cell surface and then withdrawn after a determined time interval (0.5 s) while the bending of the cantilever was observed. During this cycle, the bending of the cantilever, which is proportional to the force, was continuously measured and plotted versus tip-surface separation (i.e. distance). Two representative force-distance curves are reported in Fig. 1, when ligand/receptor interactions are not detected (black square) and when a ligand/receptor interaction is detected (red circles). At the beginning of the tip-surface approach, the cantilever deflection remains zero. Upon tip-surface contact, the cantilever bends upwards, consistent with a repulsive force that increases with the indentation. Subsequent tip-surface retraction (Fig. 1) first leads to relaxation of the cantilever bending until the repulsive force drops to zero. Upon further retraction, if the interaction between the ligand and the receptor occurs, the cantilever progressively bends downwards, reflecting an attractive force that rises with increasing tip-surface separation (Fig. 1, red circles). In every single experiment, for each time point, ∼1000 force-distances curves were performed.

### Western blot analysis

Plasma membrane-enriched fractions were prepared from 6-day cultured PC-3, HEK293 and HEK293_[SCL60]_ cells, according to the protocol reported by Limonta et al. [37]. The same procedure was used on PC-3 cells untreated or treated with LA (10^−6^ M or 10^−11^ M) for 6, 12, 18, 24 and 30 days. Samples were homogenized in 10 mM Tris-HCl (pH 7.6) buffer containing 1 mM dithiothreitol on ice. The homogenates were centrifuged two times for 10 min each at 800 *g* to remove cellular debris and the resulting supernatants were centrifuged at 18,000 *g* to pellet down the membrane fractions. The pellets were solubilized in RIPA buffer [50 mM Tris-HCl (pH 7.7), 150 mM NaCl, 0.8% Triton X-100, 0.8% sodium deoxycholate, 0.08% SDS, 10 mM EDTA, 100 µM Na_3_VO_4_, 50 mM NaF, 0.3 mM PMSF, and 5 mM iodoacetic acid]. Protein samples (100 µg/lane) were electrophoresed on a 10% polyacrylamide gel under reducing conditions. Proteins were then electroblotted onto a polyvinylidene difluoride membrane (Immobilon P, Millipore, Bedford, MA, USA) which was probed (overnight, 4°C) with the mouse monoclonal anti-GnRH-R antibody (Lab Vision Corporation, Fremont, CA, USA) (1∶100) in TBS containing 0.02% Tween 20 (TBS-T) and 5% nonfat dried milk (blocking buffer). The blot was then overlaid with the HRP-labeled secondary antibody (Vector, Burlingame, CA, USA; 1∶5,000) for 40 min in blocking buffer at room temperature. The protein bands were detected using an enhanced chemiluminescence system (ECL, Amersham, Buckinghamshire, UK) and visualised on Hyperfilm ECL (Amersham). Membranes were reprobed with an anti β-actin mAb (Sigma-Aldrich, St. Louis, MO, USA; 1∶10,000) as an internal control for protein loading. The signals were quantitated by densitometry (Chemi Doc Documentation System/Quantity One quantitation software, Bio-Rad Laboratories, Hercules, CA, USA). Densitometric units of the protein of interest were then corrected for the densitometric units of β-actin. The specific protein/β-actin ratio from each treated sample was divided by the value obtained under control conditions to obtain the fold change in GnRH-R level.

### Statistical analysis

Data were analyzed by one-way ANOVA followed by Tukey's multiple comparison test. A value of p<0.05 was considered statistically significant.

## Results

### GnRH-R quantification and binding strength in untreated PC-3 cells

**Figure 2:**
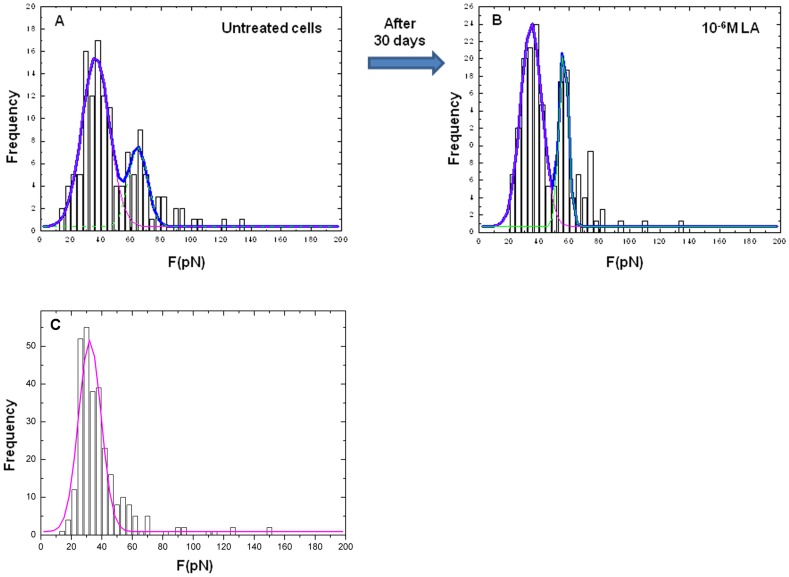
LA/GnRH-R unbinding force histograms. Distribution of unbinding events obtained at a loading rate of 2 µm/s in untreated (A) and LA (10^−6^ M)-treated (B) PC-3 cells after 30 days of culture and in HEK293_[SCL60]_ cells (C). Frequency corresponds to the number of events. In both untreated and LA-treated PC-3 cells a bimodal distribution (blue line) is clearly detected and quantified by means of two Gaussian distribution curves (purple and green dashed lines). The first peak was detected at a lower force (f∼37 pN) while the second one at a higher force (f∼65 pN). In HEK293_[SCL60]_ cells unbinding events at higher forces are less frequent.

In Fig. 2A a representative distribution of unbinding events (∼1000 force-distance cycles at a rate of 2 µm/s) obtained on untreated PC-3 cells after 6 days is shown. The bimodal distribution is characterized by the presence of two, well evident peaks, each recovered by a Gaussian fit. The first peak (purple line) was detected at a lower force (f∼37 pN) while the second one (green line) at a higher force (f∼65 pN). The presence of these two peaks suggests the existence of two distinct interactions. From the bimodal distribution, an overall unbinding probability (i.e. the probability to record an unbinding event from a single force-distance curve) of about 13% was obtained (Fig. 2A, blue line). The bimodal distribution together with the unbinding probability did not change for up to 30 days (data not shown).

To evaluate the amount of possible non-specific unbinding events occurring due to the specific tip functionalization procedure and the sensitivity of our approach, we performed the same force spectroscopy experiments on HEK293 and HEK293_[SCL60]_ cells. As expected, in wild type HEK293 cells very few tip/sample interactions were found (∼3%). On the contrary, in the GnRH-R-expressing HEK293_[SCL60]_ cells several tip/sample interactions were detected (∼59%). Interestingly, in these cells the bimodal distribution of unbinding forces was not evident since the unbinding events at higher forces (∼65 pN) are clearly less probable compared to the more frequent unbinding events at lower forces of ∼37 pN (Fig. 2C).

### GnRH-R quantification and binding strength in treated PC-3 cells

**Figure 3:**
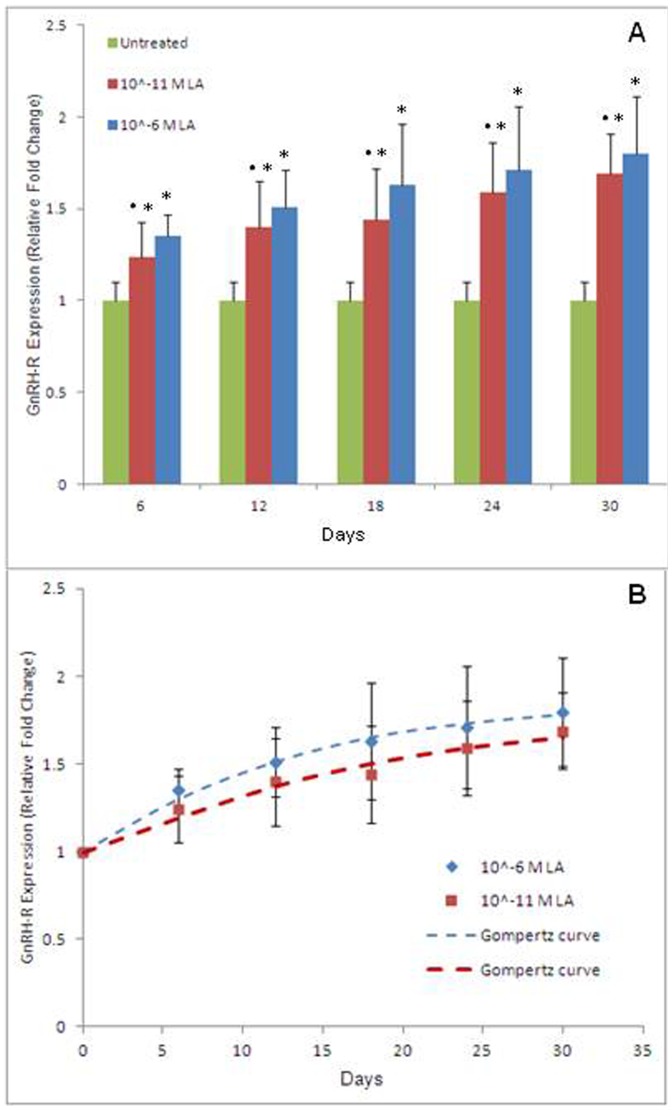
LA/GnRH-R unbinding events. (A) Histograms of LA/GnRH-R unbinding events in LA-treated PC-3 cells. In each measurement, performed every 6 days, the counting is normalized to that obtained with untreated cells (control) set to 1. Columns, mean; bars, SD. *p<0.001 *vs* control, ^•^p<0.001 *vs* 10^−6^ M LA (one-way ANOVA and Tukey's multiple comparison tests). (B) Modelling of the GnRH-R rate expression. The amount of unbinding events detected in PC-3 cells treated with 10^−6^ M LA (blue diamonds) or 10^−11^ M LA (red squares) was normalized to the amount of unbinding events in untreated cells (control) set to 1. The Gompertz curve was fitted to both the experimental data (blue or red dashed lines). The GnRH-R increase rates were clearly different [(9.3±0.1) day^−1^ for 10^−6^ M LA and (6.1±0.1) day^−1^ for 10^−11^ M LA] but tended to the same value (1.9±0.1) at long times (beyond the 30^th^ day). Data are given as mean ± SD from two independent experiments.

An increase in the agonist/GnRH-R unbinding events was observed in PC-3 cells during 30 days of exposure to LA (10^−6^ M or 10^−11^ M) (Fig. 3A). The enhancement was detectable at all the time intervals considered (from the 6^th^ to the 30^th^ day; p<0.001) and it reached the maximum value of ∼80% compared to control after 30 days of treatment with the highest dose of the analogue. A statistically significant difference was always found in the effect triggered by the two drug concentrations, with the highest dose being more effective in promoting the increase.

The same bimodal distribution (with one pick at f∼37 pN and another at f∼65 pN) detected in untreated PC-3 cells was observed in LA-treated cells over time (from the 6^th^ to the 30^th^ day), regardless the treatment administered to the cells. In Fig. 2B, a representative distribution of the unbinding events obtained at a rate of 2 µm/s in PC-3 cells treated for 30 days with 10^−6^ M LA is illustrated. The blue curve shows the bimodal distribution of the unbinding events and an overall unbinding probability of about 22%.

### Modelling of the GnRH-R rate expression on PC-3 cells

To shed light on the specific dynamic describing the increase in the exposed receptors, we compared the different amount of receptors obtained during the treatment time at the two analogue concentrations used (Fig. 3B). The increase rates were clearly different but tended to the same value at long times (beyond the 30^th^ day). This behaviour can be paralleled to those observed in tumor cells where their growth is limited by the finite space in which cells are confined and by the availability of nutrients, as modelled by the Gompertz curve [38]:
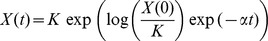
where X(t) represents the number of GnRH-R, X(0) the receptor number at the starting observation time (here normalized to one), K the maximum number of receptors that can be expressed on the cell surface and α a constant characterizing the increase rate of GnRH-R.

Figure 3B shows the best-fit curves to the experimental data obtained for PC-3 cells exposed to 10^−6^ M (red line) or 10^−11^ M (blue line) LA. In both cases, the remarkable quality of the fit allows for recovery of receptor increment rate and plateau values. The plateau value (K = 1.9±0.1) was the same at both LA concentrations used. Since K was set to 1 for untreated samples, the plateau value obtained indicates that in LA-treated PC-3 cells the receptor number can increase up to 90% compared to untreated cells, independently of the agonist concentration used. On the other hand, the increase rate was strongly influenced by LA concentration [α = (9.3±0.1) day^−1^ for 10^−6^ M LA and α = (6.1±0.1) day^−1^ for 10^−11^ M LA], thus the higher the concentration the faster the GnRH-R expression.

### Topographic distribution of the binding sites

**Figure 4:**
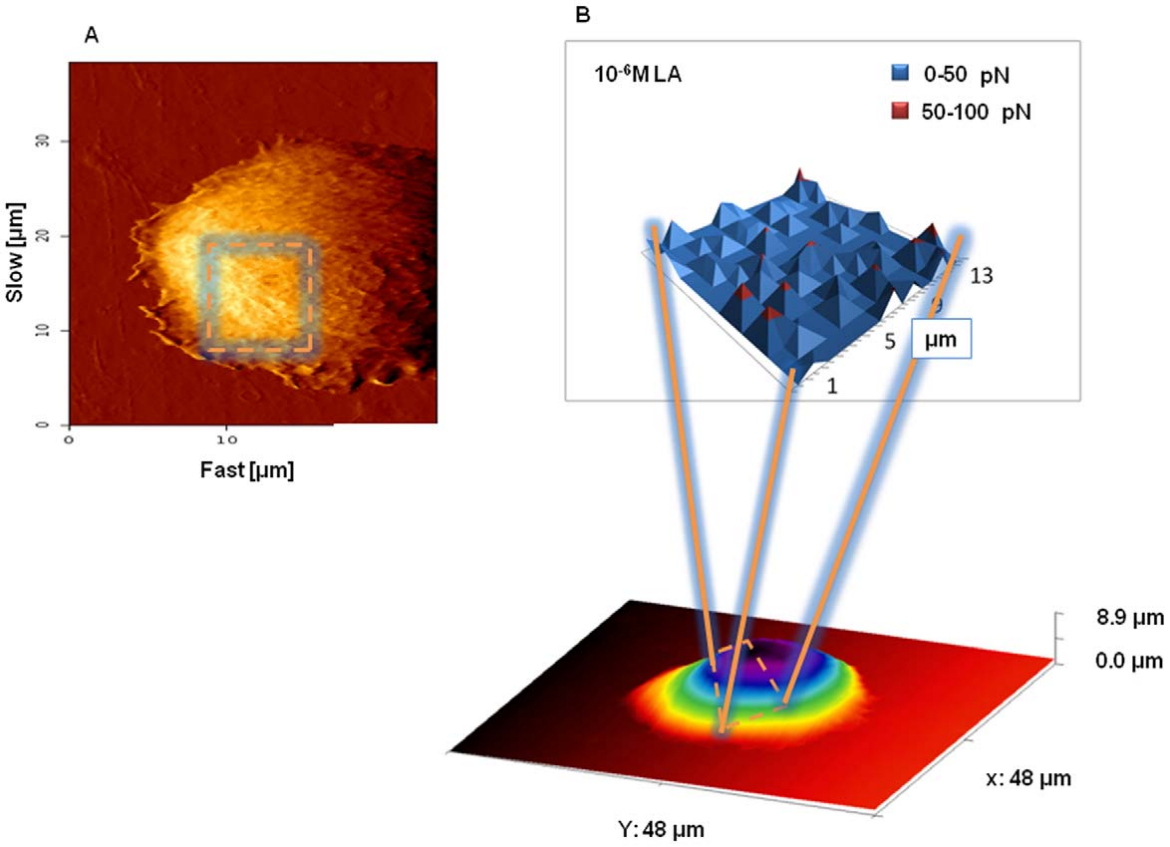
GnRH-R topography. (A) A representative high-resolution image of the PC-3 cell surface area considered for the receptor mapping. (B) A representative LA/GnRH-R unbinding force map obtained on PC-3 cells treated for 30 days with 10^−6^ M LA. In blue are represented unbinding events occurring at force <50 pN while in red those at force in the range of 50–100 pN.

**Figure 5:**
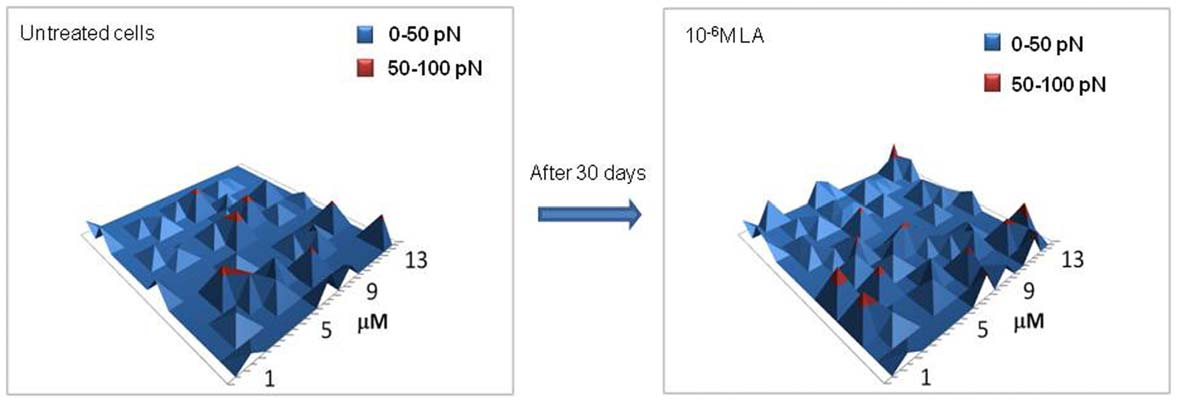
GnRH-R map of untreated and LA-treated PC-3 cells. The homogeneous distribution of the receptor molecules was not influenced by the treatment with the analogue (10^−6^ M) and did not vary through the time intervals. In blue are represented unbinding events occurring at force <50 pN while in red those at force in the range of 50–100 pN.

A bidimensional map of unbinding events performed over the cell surface furnishes a clear visualization of GnRH-R spatial distribution (Fig. 4). Unbinding events occurring at force <50 pN (corresponding to the first peak of the bimodal distribution in Fig. 2A) are represented in blue, while those in the range of 50–100 pN (corresponding to the second peak of Fig. 2A), in red. The spatial distribution of GnRH-R over the PC-3 cell surface appears homogeneous. Treatments with the agonist did not influence the receptor spatial distribution (Fig. 5) as well as the time intervals but increased the receptor level. Rarely were some clusters found (data not shown).

### GnRH-R quantification by Western blot analysis

**Figure 6:**
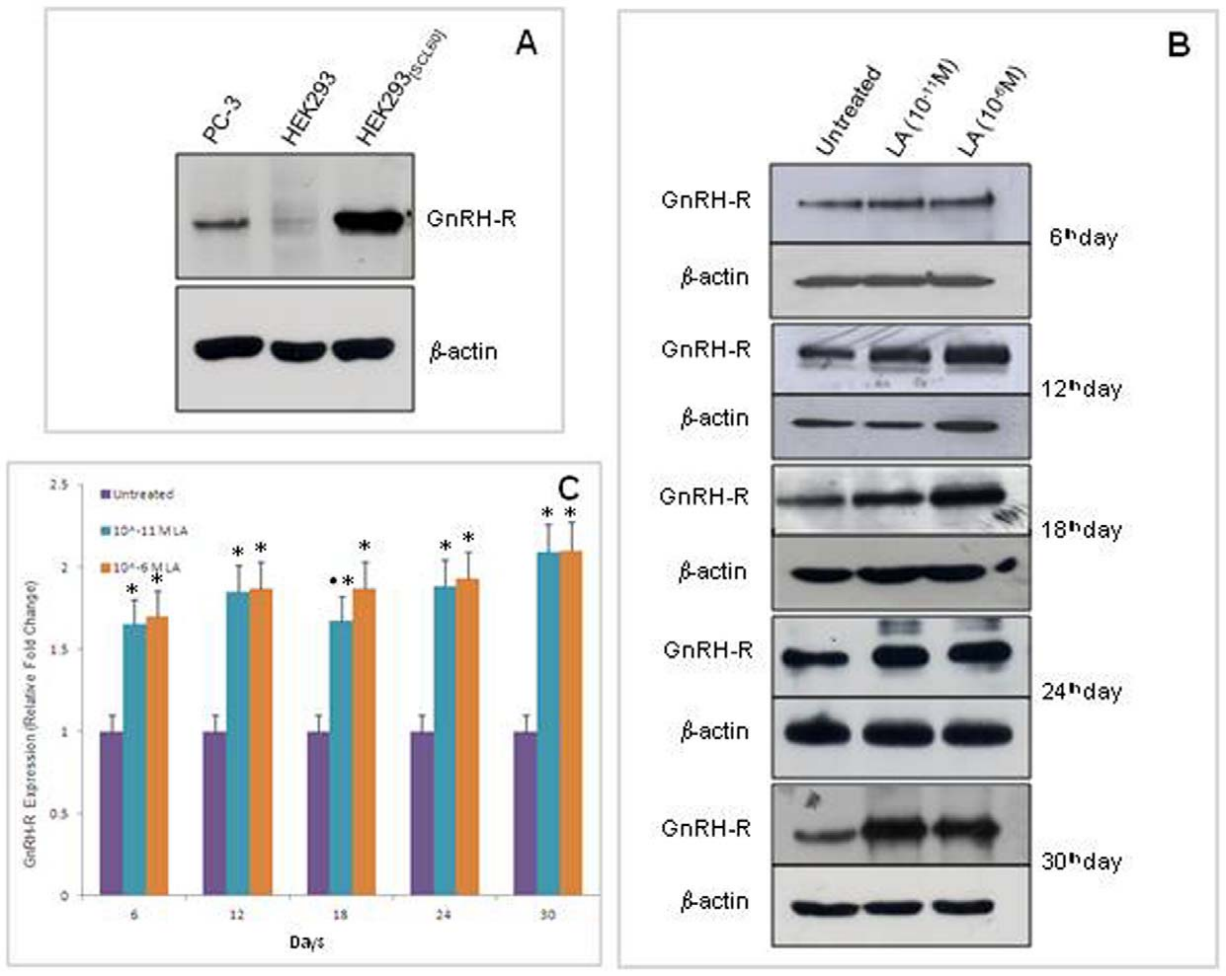
Western blot analysis of GnRH-R. (A) Western blot analysis of GnRH-R in: PC-3 cells, HEK293 cells (not expressing GnRH-R) and HEK293_[SCL60]_ cells (stably transfected with GnRH-R) after 6 days of culture. (B) Western blots showing the LA-triggered enhancement of GnRH-R in PC-3 cells treated for 6–30 days with the analogue (10^−11^ or 10^−6^ M). Representative blots from two separate experiments yielding similar results are shown. (C) Grouped densitometric data of Western blot analysis of GnRH-R. The intensity of the signals was quantified by densitometric scanning and normalized to that of β-actin (used as a loading control). Data are the ratio between values of treated and untreated samples (control, set to 1) and they are shown as mean ± SD of 2 independent experiments. *p<0.001 *vs* control, ^•^p<0.001 *vs* 10^−6^ M LA (one-way ANOVA and Tukey's multiple comparison tests).

Western blot analysis of GnRH-R-expressing cells (HEK293_[SCL60]_) revealed a clear band at approximately 60 kDa, the molecular mass of the type I pituitary receptor reported in the literature [39]. PC-3 cells showed a less intense band at the same position. A negligible signal was detected in non-transfected HEK293 cells (Fig. 6A).

In PC-3 cells, a 6–30 day treatment with both the LA concentrations (10^−11^ and 10^−6^ M) induced a statistically significant increase (from 70% to 110%, p<0.001) in the receptor levels (Fig. 6B and 6C), supporting data from atomic force spectroscopy. Even though the highest LA dose seemed to be more effective in promoting GnRH-R increase, statistical significance was not always reached (Fig. 6C).

## Discussion

The direct extrapituitary effects of GnRH analogues may be considered the result of a complex mosaic of molecular events that are necessarily dependent on the amount of GnRH-R molecules available on the cell surface and the activated signalling pathway, both of which differ among tissues [4], [40].

Scarce information is available on the effects of GnRH analogues on GnRH-R. Most of the published studies on the effects of GnRH agonists on GnRH-R expression have been carried out on *in vitro* and *in vivo* pituitary models. In these studies, the observed effects seemed to be strictly dependent on the administration modality, where a short or pulsatile exposure to the agonist led to an upregulation of the receptor levels whereas continuous and prolonged treatments determined a reduction or left them unchanged [41]–[43]. In PCa, very heterogeneous results have been reported. An immunohistochemical study described a GnRH-R decrease in PCa samples from patients who underwent a 3-month long neoadjuvant hormonal therapy with leuprolide and the antiandrogen bicalutamide, compared to the immunoreactivity observed in samples from untreated patients [44]. This finding was attributed by the authors to the binding of the analogue to PCa cells. Also in DU-145 xenografted PCas, an agonist-induced slight decrease in the GnRH-R levels was described, while no significant variations were observed in the receptor mRNA levels [45]. Similar results were obtained when PCa cells and fibroblasts, isolated from patients' samples, were cocultured and exposed to leuprolide [46]. On the other hand, a strong increase in GnRH-R mRNA was described in rat prostate after 28 days of treatment with goserelin [43]. In this context, we observed by Western blotting that LA was able to induce a post-transcriptional upregulation of membrane GnRH-R after 4, 6 and 12 days of treatment in androgen-insensitive, highly invasive and poorly differentiated PC-3 cells [24].

With the aim of gaining insight into the functional properties of GnRH-R exposed on cell surface, and therefore actively involved in the agonist-receptor signalling, an AFM-based approach on living PC-3 cells was adopted for the first time in the present study. The effects of a long and continuous treatment with LA on crucial aspects of the membrane GnRH-R (i.e. quantity of the unbinding events, strength of the analogue-receptor binding and receptor distribution) were investigated.

By detecting the amount and the strength of the interaction, we demonstrated the ability of the hormone to increase the expression/availability of GnRH-R at the PC-3 cell surface, when continuously administered to the cells, with a maximum increase of ∼80% after 30 days of treatment with the highest dose of LA.

In this context, we also studied the kinetics of GnRH-R exposure in PC-3 cells treated with high and low agonist concentrations. Kinetics was analyzed by fitting the Gompertz curve, a logistic curve widely adopted to describe the population increase in several biologic systems. The Gompertz curve well recovers the time-dependent increase of the GnRH-R at the cell surface. According to this model, the rate of expression of GnRH-R in LA-treated cells depends on the agonist concentration used, whereas the asymptotic value is independent of the LA concentration and related to the specific cell characteristics and experimental condition used. This evidence, while clearly establishing the occurrence of an LA-triggered long-lasting upregulation of its own receptor at the cell surface, allows for the detection of the maximum amount of receptors that the cell can express. This latter finding enables the fine-tuning of the dose/effect ratio by changing LA concentration. In accordance with the above results are data from immunoblot analysis of membrane GnRH-R performed on PC-3 cells treated for 6–30 days with the analogue, which clearly showed LA efficacy in inducing a significant upregulation of the receptor, as previously demonstrated by the same technique until 12 days of treatment [24]. Western blot findings agree with AFM data as the highest LA dose seemed to be more efficacious in promoting GnRH-R increase, although statistical significance was not always reached. This may be due to the intrinsic differences between the two methods.

As for the mechanisms responsible for the analogue-induced receptor enhancement, various functional parameters may be involved in determining the amount of receptors available at the plasma membrane. Our previous study regarding the LA-induced upregulation of GnRH-R at the PC-3 cell surface demonstrated that such effect occurred at a post-transcriptional level [24]. Thus, it is conceivable that the agonist may act increasing the translation rate of the receptor transcript and/or slowing protein degradation. On the basis of literature, it is also possible to speculate that one of the involved mechanisms may be represented by agonist ability to promote the GnRH-R exit from the endoplasmic reticulum (ER) therefore favouring the receptor anterograde trafficking, within intracellular transport vesicles, to the plasma membrane. This would be consistent with the observation that other seven transmembrane (7TM) receptors, such as δ-opioid peptide receptors, that are widely stored within ER, are sent to the cell surface in response to activation of a small subpopulation of membrane receptors [47]. Since 7TM receptors also undergo retrograde transport from the cell surface via endosomes, an alternative possible explanation is that GnRH-R activation could inhibit receptor internalization, that for type I mammalian GnRH-R is known to occur very slowly [48].

The persistence of high receptor levels at the cell surface of androgen-insensitive cells may warrant the maintenance of their response to the agonist treatment due to the direct activity of the hormone. Moreover, accessibility of receptor molecules at the cell surface may allow for the development of new therapeutic strategies (i.e. targeted therapies), if confirmed by studies including other androgen-unresponsive cell lines. However, it remains to be established whether a similar mechanism also operates *in vivo*. In this regard, a clear correlation between the degree of expression of GnRH-R at the cell surface and the extent of the agonist-induced antiproliferative response has been demonstrated both *in vitro* and *in vivo* [25]. It has even been hypothesized the usefulness of patients' screening and phenotyping in order to select those who express high levels of GnRH-R at the tumor cell membrane and thus may benefit from the direct activity of the agonists [49]. The LA-induced receptor enhancement observed in our study occurred not only with a high concentration of the analogue but also when a very low dose (10^−11^ M) was used. In this regard, it is worth mentioning that in PCa patients who undergo subcutaneous administration of slow-release preparation of the analogue the drug plasma concentration reaches values around 10^−9^ M [50].

The unbinding force histograms, recovered by single molecule force spectroscopy, yield further insights into the biochemical nature of the GnRH-R. Indeed, the bimodal distribution of the analogue/GnRH-R interactions in androgen-insensitive PC-3 cells could be ascribed to several causes: the stochastic presence of non-specific forces, the simultaneous binding of more than one receptor, by accidental different orientations of the pulling direction, or it may suggest the existence of two kinds of receptor. The latter appears to be the most intriguing since it seems to indicate the existence of two classes of GnRH-R characterized by two different binding affinities. Because of the occurrence of a large number of unbinding events at a lower force, lower affinity binding sites are prevalent. This finding is in partial agreement with data from other authors who found a single class of low affinity binding sites in PC-3 cells [51].

Finally, a homogeneous distribution of the receptor molecules on the PC-3 cell surface was observed which was not influenced by the treatment with the analogue and did not vary through the different time intervals. It has been reported that the GnRH agonist-receptor complex is initially uniformly distributed on the surface of the gonadotrope cells [52]. In fact, patching, capping, microaggregation and internalization of the agonist-receptor complex begin only a few minutes after the receptor occupancy. The topographical distribution of the receptors we found on the PC-3 cell surface is consistent with the fact that only the unbound fraction of the GnRH-R population has been evaluated by AFM.

In conclusion, our study provides a crucial insight into the stimulatory effect of LA on GnRH-R expressed on the androgen-insensitive PCa cell membrane. The information concerning the ability of the analogue to induce a long-lasting upregulation of its own receptor at the cell surface may encourage further studies to verify the possibility of using the analogue in the treatment of androgen-unresponsive PCa patients by means of its direct activity.

Moreover, this study highlights the fact that AFM, for its capability to work on living cells and to detect single molecule interactions, is a powerful tool to investigate important features of the poorly understood agonist/receptor interaction that could be used to address structural/chemical analogue optimizations.
